# Evaluation of the hypoglycaemic and antioxidant effects of submerged *Ganoderma lucidum* cultures in type 2 diabetic rats

**DOI:** 10.1080/21501203.2020.1733119

**Published:** 2020-03-01

**Authors:** Chung-Hsiung Huang, Wei-Kang Lin, Shun‐Hsien Chang, Guo-Jane Tsai

**Affiliations:** a Department of Food Science, National Taiwan Ocean University, Keelung, Taiwan; b Institute of Food Safety and Risk Management, National Taiwan Ocean University, Keelung, Taiwan; c Center for Marine Bioscience and Biotechnology, National Taiwan Ocean University, Keelung, Taiwan

**Keywords:** Antioxidant, *Ganoderma lucidum*, hypoglycaemic effect, submerged culture, type 2 diabetes

## Abstract

We aim to investigate the hypoglycaemic and antioxidant effects of submerged *Ganoderma lucidum* cultures and elucidate the potential mechanisms behind these effects using a type 2 diabetic rat model. Diabetic rats were daily fed with a high-fat diet supplemented with 1% or 3% freeze-dried whole submerged cultures of *G. lucidum* or mycelia for 5 weeks. We observed significantly decreased fasting plasma glucose levels, homoeostasis model assessment equation-insulin resistance, and plasma glucose in oral glucose tolerance test. Furthermore, we observed increased levels of glycogen, hepatic hexokinase, glucose-6-phosphate dehydrogenase, and intestinal disaccharidase activities. *G. lucidum* supplement downregulated the plasma levels of aspartate aminotransferase, alanine aminotransferase, creatinine, and urea nitrogen as well as liver and kidney levels of thiobarbituric acid reactive substances. Based on the hypoglycaemic and antioxidant effects of *G. lucidum* submerged cultures, we recommend the potential application of these products as functional foods or additives for controlling type 2 diabetes.

**Abbreviations** ALT: Alanine aminotransferase; AST: Aspartate aminotransferase; BUN: Blood urea nitrogen; BW: Body weight; CREA: Creatinine; FPG: Fasting plasma glucose; G6Pase: Glucose-6-phosphatase; G6PD: Glucose-6-phosphate dehydrogenase; HOMA-IR: Homoeostasis model assessment of insulin resistance; OGTT: Oral glucose tolerance test; PTP: Protein tyrosine phosphatase; STZ: Streptozotocin; TBARS: Thiobarbituric acid reactive substances

## Introduction

*Ganoderma lucidum*, a white rot basidiomycete that grows on logs, is one of the most famous traditional Chinese medicines and has been used as a food supplement in East Asia for centuries (Wachtel-Galor et al. 2011). The fruiting bodies of *G. lucidum* possess numerous biological activities, including anti-tumour, immune modulatory, hepatoprotective, antioxidant, anti-ageing, and hypoglycaemic effects (Wachtel-Galor et al. 2011; Girometta 2019). Although fruiting bodies are produced naturally, they are not produced in sufficient quantities for commercial use (Wachtel-Galor et al. 2011). The artificial cultivation of fruiting bodies takes approximately 3–5 months. In contrast, submerged ganoderma cultures only take 2–3 weeks to produce large amounts of bioactive ingredients such as mycelium, polysaccharides, and ganoderic acid. Generally, the advantages of submerged cultures compared to traditional basidiocarp cultivation include lower production and time costs, less space requirement, easier control of environmental conditions, and higher yields, purity, and rejuvenation (Wachtel-Galor et al. 2011).

Type 2 diabetes, a long-term metabolic disorder characterised by high plasma glucose levels and insulin resistance, mainly caused by obesity and lack of exercise, accounting for approximately 90% of diabetes cases (Wu et al. 2014). Long-term complications of high plasma glucose levels include heart diseases, stroke, and diabetic retinopathy, which can cause blindness, kidney failure, and poor limbic blood flow, possibly leading to amputations (Olokoba et al. 2012). Diabetes is diagnosed by blood tests, such as fasting plasma glucose (FPG) test, oral glucose tolerance test (OGTT), and homoeostasis model assessment of insulin resistance (HOMA-IR)(Boyko et al. 2007). To date, type 2 diabetes has been controlled through weight loss, healthy eating, regular exercise, diabetes medication or insulin therapy, and blood sugar monitoring (Olokoba et al. 2012). Nevertheless, these strategies are just designed for the retardation of development or progression of complications.

It has been well known that hyperglycaemia in patients with type 2 diabetes is not only associated with insulin resistance but also with endogenous glucose production. Hepatic control of endogenous glucose homoeostasis is achieved *via* the coordination of signalling pathways that regulate glycogen synthesis, glycogenolysis, and gluconeogenesis (Sloop et al. 2007). Glucose-6-phosphatase (G6Pase) and hexokinase are key regulators for endogenous glucose production, and the reduction of G6Pase/hexokinase ratio might improve glucose control in type 2 diabetes (Sloop et al. 2007). Diabetes and the deficiency of glucose-6-phosphate dehydrogenase (G6PD), the major enzyme that catalyzes the first step of the pentose phosphate pathway, have been reported to aggravate each other, and a possible association has been reported between diabetes and G6PD deficiency (Heymann et al. 2012). On the other hand, intestinal disaccharidase, a critical enzyme involved in the digestion of disaccharides into glucose, is also important for the regulation of plasma glucose levels (Neyrinck et al. 2016). Collectively, enzymes related to glucose absorption or metabolism are also potential targets for developing therapeutic strategies for hyperglycaemia.

Hyperglycaemia in diabetes is a key mechanism leading to oxidative stress, which is related to organ damage. Oxidative stress is one of the major pathogenic mechanisms involved in the progression of diabetes and diabetic complications, such as liver and kidney damage (Raza et al. 2011). Moreover, lipid peroxidation-mediated tissue damage has been detected during the progression of diabetes, which is also one of the specific features of chronic diabetes (Manna et al. 2010). Therefore, natural products with antioxidant activity are important because they can potentially improve the oxidative complications associated with diabetes.

Although several studies have reported the hypoglycaemic and antioxidant potentials of *G. lucidum* fruiting bodies (He et al. 2006; Seto et al. 2009; Xiao et al. 2012; Zheng et al. 2012; Pan et al. 2013; Bach et al. 2018), the composition and relative proportions of *G. lucidum* basidiocarp cultivation are different from those of submerged culture (Wachtel-Galor et al. 2011). In addition, the yield of mycelia and extracellular polysaccharides obtained from submerged *G. lucidum* cultures varies with the culture conditions (Chang et al. 2006). Therefore, it would be of great interest to know whether the submerged *G. lucidum* cultures also possess hypoglycaemic and antioxidant effects. In the present study, we used a type 2 diabetic rat model to deepen our understanding of the hypoglycaemic and antioxidant effects of submerged *G. lucidum* culture, and to explore its mechanism of action. First, we measured the contents of mycelium and extracellular polysaccharides in the submerged *G. lucidum* cultures. Next, the STZ-induced diabetic rats were daily fed with a high-fat diet supplemented with 1% or 3% freeze-dried whole submerged *G. lucidum* cultures (1 G or 3 G) or mycelia (1 M or 3 M) for 5 weeks. FPG and HOMA-IR levels were measured, and OGTT was conducted in rats fed with a *G. lucidum*-supplemented diet to explore its hypoglycaemic effects. Liver glycogen levels and hepatic hexokinase, G6Pase, G6PD, and intestinal disaccharidase activities were evaluated to illustrate the potential mechanism of action behind the antioxidant effects of submerged *G. lucidum* cultures. Finally, the levels of aspartate aminotransferase (AST), alanine aminotransferase (ALT), creatinine (CREA), and urea nitrogen (BUN) in plasma as well as levels of thiobarbituric acid reactive substances (TBARS) in the liver and kidney were measured to investigate the antioxidant and protective effects of *G. lucidum* against diabetes-related organ damage.

## Materials and methods

### Chemicals and reagents

All chemicals and reagents were purchased from Sigma-Aldrich Chemical Co. (St. Louis, MO, USA) unless otherwise stated. The rat insulin enzyme-linked immunosorbent assay (ELISA) kit was purchased from Randox Laboratories, Ltd. (Crumlin, UK), and the glucose detection kit was purchased from Audit Diagnostics (Cork, Ireland).

### Preservation, culture and sample preparation

*G. lucidum* BCRC 36123 purchased from the Bioresources Collection and Research Centre (Hsinchu, Taiwan) was maintained on Gano medium (2.4 g glucose, 0.6 g yeast extract in 100 mL deionised water) agar plates and cultured at 30°C for 7 days. Next, pieces of mycelium pad (0.5 x 0.5 cm^2^) were cut from the culture plate and inoculated into preserving medium at room temperature for 2 h followed by overnight storage at 4°C. The liquid cultures were then stored at −80°C. For culturing, the frozen pieces were thawed at 37°C and cultured on Gano medium agar plates at 30°C for 7 days. Two mycelial pieces were cut from the culture plate and inoculated into a flask containing Gano medium and incubated at 30°C with shaking at 110 rpm for 7 days. Finally, based on the methods in our previous report (Chang et al. 2006), the cultivated mycelia were separated by centrifugation. After being thoroughly washed, the collected precipitated mycelia were freeze-dried and weighed. The extracellular polysaccharides in supernatant were precipitated by ethanol, washed, and finally freeze-dried and weighed. Whole submerged cultures and precipitated mycelia were freeze-dried and stored at −20°C for animal experiments.

### Animals and experimental design

Eight-week-old male Sprague-Dawley rats were obtained from BioLASCO Taiwan Co., Ltd. (Taipei, Taiwan). On arrival, rats were housed in stainless steel cages with diet and water provided *ad libitum* for 1 week before treatment. Rats were kept under humid conditions (30%–70%) and 12-h light/dark cycle in a temperature (23°C ± 2°C)-controlled environment. All animal experiments were performed in accordance with the guidelines for the care and use of laboratory animals and approved by the Institutional Animal Care and Use Committee of the National Taiwan Ocean University.

**Figure 1.**
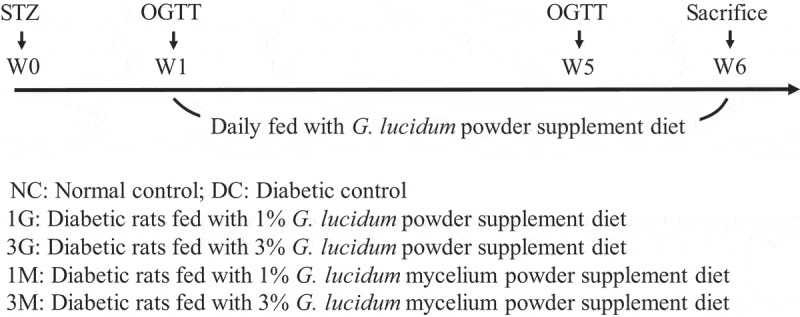
Protocols of diabetes induction and *G. lucidum* treatment. Sprague-Dawley rats were injected with nicotinamide and streptozotocin (STZ) to induce type 2 diabetes. After 1 week, oral glucose tolerance test (OGTT) was performed to verify successful induction of diabetes. The rats in normal control (NC) group did not receive any treatment through the experiment. Diabetic rats were randomly divided to 5 groups (n = 8): diabetic control (DC); diabetic + 1% G (1 G); diabetic + 3% G (3 G); diabetic + 1% M (1 M) and diabetic + 3% M (3 M). Except for the rat in NC and DC groups, the other rats were daily fed with *G. lucidum* powder supplement diets as described in Materials and methods. OGTT was performed again at week 5. All rats were weighted and sacrificed at week 6 to harvest the samples of blood, liver and small intestine for further analysis

The rats were randomly divided into six groups (n = 8): normal control (NC), diabetic control (DC), diabetic rats fed with 1% or 3% freeze-dried whole submerged *G. lucidum* culture-supplemented diets (1 G or 3 G), and diabetic rats fed with 1% or 3% freeze-dried *G. lucidum* mycelia-supplemented diets (1 M or 3 M). For the induction of type 2 diabetes, the rats were subcutaneously injected with nicotinamide (230 mg/kg BW) and STZ (65 mg/kg BW) with a 15-min interval (Masiello et al. 1998). After 1 week, OGTT was performed to confirm the successful induction of diabetes, and the rats were daily fed with a *G. lucidum* supplement diet for 5 weeks. The intake levels of food and water, body weight, urine volume, and faecal weight were measured weekly. OGTT was performed again at the fourth week after diabetes induction, and blood, liver, and intestinal tissue samples were harvested after sacrifice of the rats at the fifth week for further analysis (Figure 1).

### Evaluation of the plasma levels of glucose, insulin, AST, ALT, CREA, and BUN

Blood samples were collected for the preparation of plasma at the designated time points after the beginning of OGTT and before sacrifice. The plasma glucose, insulin, AST, ALT, CREA, and BUN levels were measured using a glucose detection kit, insulin ELISA kit, and Enzymatic Kits (Randox Laboratories Limited, UK), respectively, according to the manufacturer’s instructions. The HOMA-IR was calculated using the equation described in the previous study (Boyko et al. 2007).

### Evaluation of the levels of glycogen, hexokinase, glucose-6-phosphatase, and glucose-6-phosphate dehydrogenase in the liver

Liver glycogen level was measured and calculated as described previously (Murat and Serfaty 1974). Briefly, liver samples were homogenised in citrate buffer/tri-sodium citrate solution. After centrifugation, the supernatant was harvested, and the glucose level was measured at 505 nm using a glucose detection kit. The other part of supernatant was mixed with amyloglucosidase and incubated at 37°C for 4 h. After centrifugation, the supernatant was harvested, and the glucose level was measured to calculate the concentration of glycogen. In order to determine the activities of hepatic enzymes pertaining to the control of endogenous glucose homoeostasis, liver samples were homogenised in *N*-acetyl-cysteine buffer and centrifuged to obtain the cytosol of hepatocytes. Hexokinase, G6Pase, and G6PD activities in the cytosol of hepatocytes were measured as described previously (Erickson et al. 1978).

### Evaluation of disaccharidase activity in the intestinal mucosal tissues

The activity of intestinal disaccharidase, including maltase, lactase and sucrose, was measured based on the method described by Dahlqvist (1968). Briefly, the mucosal tissues of small intestine were harvested and homogenised in maleic acid buffer. After centrifugation, the supernatant was harvested and mixed with either maltose, lactose, or sucrose. The mixtures were incubated at 37°C for 60 min and then 100°C for 2 min to completely inactivate disaccharidase. Glucose and protein levels were measured using a glucose detection kit and Bio-Rad protein assay, respectively, according to the manufacturer’s instructions.

### Evaluation of TBARS levels in the liver and kidney

Liver and kidney samples were homogenised as described in the previous section. Homogenised samples were mixed with phosphoric acid and thiobarbituric acid and incubated at 95°C for 45 min. After cooling down, the samples were mixed with n-butanol and centrifuged to collect the supernatants for the measurement of optical density.

### Statistical analysis

All data were expressed as the mean ± standard error of mean for each treatment group. Statistical analysis was performed by SPSS 12.0 software, and independent sample *t*-test was used to assess the statistical difference between treatment groups and the DC control. All *p*-values < 0.05 were considered statistically significant.

## Results

### Mycelia and polysaccharide levels in the submerged G. lucidum cultures and the intake doses of G. lucidum

After incubation of G. lucidum at 30°C for 7 days, the amounts of mycelia and extracellular polysaccharides in culture were measured (Chang et al. 2006). As shown in Table 1, mycelia and extracellular polysaccharide levels were 6.56 ± 0.74 g/L and 0.50 ± 0.08 g/L, respectively. The pH value of the cultured medium was decreased from 6.24 ± 0.05 at the beginning to 3.90 ± 0.07 at the end of incubation (Table 1).

**Table 1.** Contents of mycelium, extracellular polysaccharides and pH value in the submerged culture of *Ganoderma lucidum* at 30°C for 7 days

Item	pH value (before)	pH value (after)	Mycelium (g/L)	Extracellular Polysaccharides (g/L)
*Ganoderma lucidum*	6.24 ± 0.03	3.90 ± 0.04	6.56 ± 0.43	0.50 ± 0.05

Results are expressed as mean ± SEM for *Ganoderma lucidum* (n = 3).

Based on the food intake and body weights of rats (Tables 2 and 3), the intake doses of mycelia were calculated as 0.5, 1.5, 0.46, and 1.39 g/kg/day for rats in the 1 G, 3 G, 1 M, and 3 M groups, respectively. Extracellular polysaccharide intake was calculated as 36 and 107 mg/kg/day for rats in the 1 G and 3 G groups, respectively.

**Table 2.** The average daily food intake, drinking water intake, urine volume and faeces weights of rats fed with *G. lucidum*-containing high- cholesterol diets

Diet	NC	DC	1 G	3 G	1 M	3 M
Food intake (g/day)	23.2 ± 0.39	23.0 ± 0.42	23.8 ± 0.40	23.8 ± 0.42	22.4 ± 0.55	21.9 ± 0.61
Water drink (g/day)	27.7 ± 2.22	29.4 ± 2.72	27.1 ± 1.78	29.6 ± 1.97	29.6 ± 2.87	27.7 ± 2.77
Urine volume (mL/day)	16.8 ± 3.31	16.7 ± 2.90	14.5 ± 2.71	18.5 ± 3.25	15.6 ± 3.06	12.8 ± 2.61
Faeces wet weight (g/day)	2.12 ± 0.23	1.80 ± 0.10	2.12 ± 0.13	2.13 ± 0.13	2.07 ± 0.07	1.93 ± 0.05
Faeces dry weight (g/day)	1.69 ± 0.13	1.64 ± 0.08	1.81 ± 0.10	1.86 ± 0.09	1.79 ± 0.06	1.68 ± 0.03

Results are expressed as mean ± SEM for each group of rats (n = 8). p < 0.05 compared with the normal control (NC); *p < 0.05 compared with diabetic control (DC).

**Table 3.** The body weights, small intestine, liver and kidney weights of diabetic rats fed with *G. lucidum*-containing high-cholesterol diets

Diet	NC	DC	1 G	3 G	1 M	3 M
Initial body weight (g)	347 ± 9.2	347 ± 10.5	340 ± 7.5	349 ± 5.8	352 ± 9.4	353 ± 6.8
Final body weight (g)	463 ± 9.9	472 ± 8.7	456 ± 12.1	464 ± 4.8	459 ± 8.2	458 ± 6.8
Small intestine weight (g)	10.2 ± 0.31	9.10 ± 0.49	9.35 ± 0.58	9.25 ± 0.44	9.72 ± 0.46	9.28 ± 0.43
Liver weight (g)	20.1 ± 0.52	21.5 ± 1.12	22.0 ± 1.07	23.0 ± 1.05	21.4 ± 1.24	20.7 ± 0.85
Kidney weight (g)	3.05 ± 0.18	3.11 ± 0.14	2.88 ± 0.06	3.03 ± 0.16	3.09 ± 0.12	2.90 ± 0.17

Results are expressed as mean ± SEM for each group of rats (n = 8). p < 0.05 compared with the normal control (NC); *p < 0.05 compared with the diabetic control (DC).

### Diets supplemented with G. lucidum reduced the plasma glucose levels

**Figure 2.**
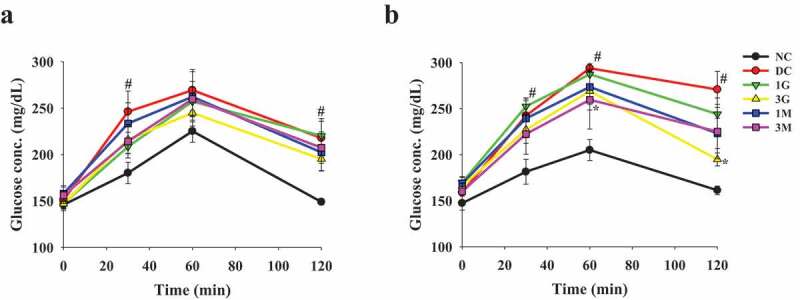
The concentration of plasma glucose in rats before and after *G. lucidum* powder supplement in oral glucose tolerance test (OGTT). The rats were injected with nicotinamide and STZ for the induction of diabetes and fed with *G. lucidum* powder supplement diets for 5 weeks as described in **Materials and methods**. (a) Before and (b) after *G. lucidum* powder supplement for 4 weeks, the blood samples were collected at 0, 30, 60 and 120 min after glucose administration. The concentration of plasma glucose was measured by enzymatic kit following the supplier’s instruction. Results are expressed as mean ± SEM for each group of rats (n = 8). ^#^*p* < 0.05 compared with the NC group; *p < 0.05 compared with the DC group. NC: normal control; DC: diabetic control; 1 G: diabetic rats fed with 1% *G. lucidum* powders; 3 G: diabetic rats fed with 3% *G. lucidum* powders; 1 M: diabetic rats fed with 1% *G. lucidum* mycelium powders. 3 M: diabetic rats fed with 3% *G. lucidum* mycelium powders

**Figure 3.**
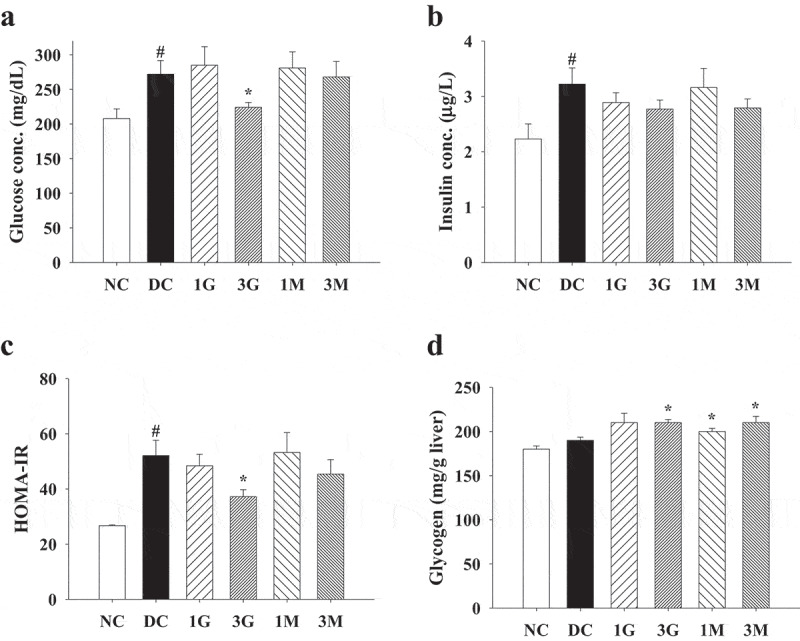
The levels of plasma glucose parameters and glycogen concentrations in liver of diabetic rats fed with *G. lucidum* powder supplement diets. The rats were injected with nicotinamide and STZ for the induction of diabetes and fed with *G. lucidum* powder supplement diets for 5 weeks. The blood samples were collected, and rats were sacrificed to harvest liver samples. The levels of (a) glucose and (b) insulin in plasma and (d) glycogen in liver were detected, and the value of (c) homoeostasis model assessment equation-insulin resistance (HOMA-IR) was calculated as described in **Materials and methods**. Results are expressed as mean ± SEM for each group of rats (n = 8). ^#^*p* < 0.05 compared with the NC group; *p < 0.05 compared with the DC group. NC: normal control; DC: diabetic control; 1 G: diabetic rats fed with 1% *G. lucidum* powders; 3 G: diabetic rats fed with 3% *G. lucidum* powders; 1 M: diabetic rats fed with 1% *G. lucidum* mycelium powders. 3 M: diabetic rats fed with 3% *G. lucidum* mycelium powders

In order to confirm the induction of diabetes, OGTT was performed 1 week after STZ injection. After oral administration with glucose, the plasma glucose level was markedly elevated in rats injected with STZ compared with that in the NC group, indicating successful induction of diabetes (Figure 2(a)). OGTT was performed to investigate the hypoglycaemic effects of *G. lucidum* after feeding the rats with *G. lucidum* supplement diets for 4 weeks. Although the plasma glucose level was only slightly lower in rats in the 1 G, 1 M, and 3 M groups, it was significantly reduced in the 3 G group compared with that in the DC group (Figure 2(b)). In order to confirm the hypoglycaemic effects of the *G. lucidum* supplement diets, the FPG levels and liver glycogen concentration were measured. Consistent with the results obtained from OGTT, the FPG levels in the 3 G group, but not those in other treatment groups, was significantly reduced compared with that in the DC group (Figure 3(a)). In parallel, similar trend of plasma insulin levels were observed for each group (Figure 3(b)). As HOMA-IR can more consistently predict type 2 diabetes compared with other insulin resistance indices, we further calculated HOMA-IR based on FPG and insulin levels (Boyko et al. 2007). We observed that 3 G treatment significantly reduced HOMA-IR (Figure 3(c)). Furthermore, the glycogen level in the liver was significantly increased in the 3 G, 1 M, and 3 M groups (Figure 3(d)). Accordingly, we suggest the potential involvement of carbohydrate metabolism in the hypoglycaemic effect of *G. lucidum*.

### G. lucidum supplement diets modulated the activities of hepatic carbohydrate metabolism enzymes

**Figure 4.**
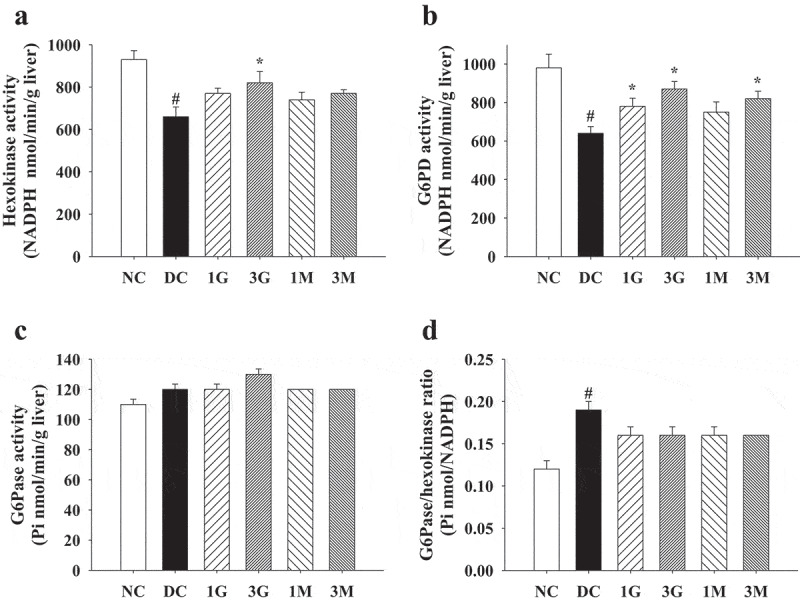
The activities of carbohydrate metabolism enzymes in liver of diabetic rats fed with *G. lucidum* powder supplement diets. The rats were injected with nicotinamide and STZ for the induction of diabetes and fed with *G. lucidum* powder supplement diets for 5 weeks. The rats were sacrificed, and the liver samples were harvested and prepared for the detection of (a) hexokinase, (b) glucose-6-phosphate dehydrogenase (G6PD) and (c) glucose-6-phosphatase (G6Pase) activities and calculation of (d) G6Pase/hexokinase ratio as described in **Materials and methods**. Results are expressed as mean ± SEM for each group of rats (n = 8). *p* < 0.05 compared with the NC group; *p < 0.05 compared with the DC group. NC: normal control; DC: diabetic control; 1 G: diabetic rats fed with 1% *G. lucidum* powders; 3 G: diabetic rats fed with 3% *G. lucidum* powders; 1 M: diabetic rats fed with 1% *G. lucidum* mycelium powders. 3 M: diabetic rats fed with 3% *G. lucidum* mycelium powders

Based on the above results, we further elucidated the influence of *G. lucidum* supplement diets on the activities of carbohydrate metabolism enzymes. Hexokinase and G6PD activities in rat livers in the DC group were significantly reduced compared with those in the NC group (Figure 4 (a,b)). However, *G. lucidum* supplement diets, particularly 3 G, could reverse the diminished activities of hexokinase and G6PD (Figure 4 (a,b)). Although G6Pase activity was comparable between each group, the G6Pase/hexokinase ratio was reduced in rats fed with *G. lucidum* supplement diets compared with that in the DC group (Figure 4 (c,d)). These findings suggest that modulation of carbohydrate metabolism enzyme activities is one of the major mechanisms behind the hypoglycaemic effect of *G. lucidum*.

### G. lucidum supplement diets ameliorated diabetes-associated intestinal damage

**Figure 5.**
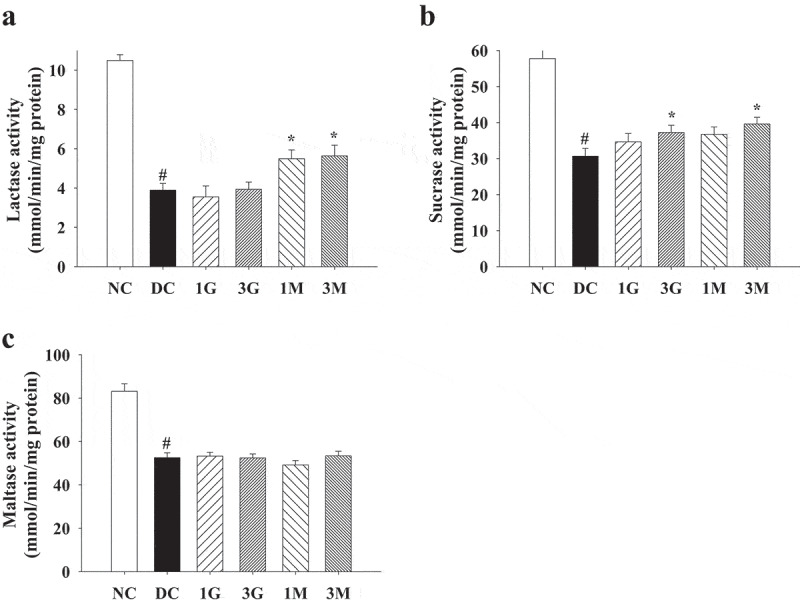
The activities of small intestinal disaccharidases in diabetic rats fed with *G. lucidum* powder supplement diets. The rats were injected with nicotinamide and STZ for the induction of diabetes and fed with *G. lucidum* powder supplement diets for 5 weeks. The rats were sacrificed, and the tissues of small intestine were harvested and prepared to evaluate the activities of (a) lactase, (b) sucrase and (c) maltase as described in Materials and methods. Results are expressed as mean ± SEM for each group of rats (n = 8). *p* < 0.05 compared with the NC group; *p < 0.05 compared with the DC group. NC: normal control; DC: diabetic control; 1 G: diabetic rats fed with 1% *G. lucidum* powders; 3 G: diabetic rats fed with 3% *G. lucidum* powders; 1 M: diabetic rats fed with 1% *G. lucidum* mycelium powders. 3 M: diabetic rats fed with 3% *G. lucidum* mycelium powders

In some cases of chronic diabetes, the enteric nerves supplying in the small intestine might be affected, leading to abnormal motility, secretion, or absorption (Wolosin and Edelman 2000). Decreased intestinal absorption of glucose would stimulate glycogenolysis in the liver and subsequently result in hyperglycaemia (Pan et al. 2013). Therefore, we also investigated the changes in small intestinal weight and the activities of disaccharidases in the small intestine. Small intestinal weight was slightly reduced in STZ-injected rats compared with that in the NC group (Table 3). The *G. lucidum* supplement diets did not induce significant alterations in the small intestinal weight (Table 3). Notably, maltase, lactase, and sucrase activities were markedly reduced in STZ-injected rats. However, the *G. lucidum* supplement diets significantly reversed the reduction in lactase and sucrase activities (Figure 5 (a,b)). These findings suggested a potential protective effect of *G. lucidum* supplement diets against diabetes-induced intestinal damage.

### G. lucidum supplement diets attenuated diabetes-associated oxidative damage in the liver and kidney

**Figure 6.**
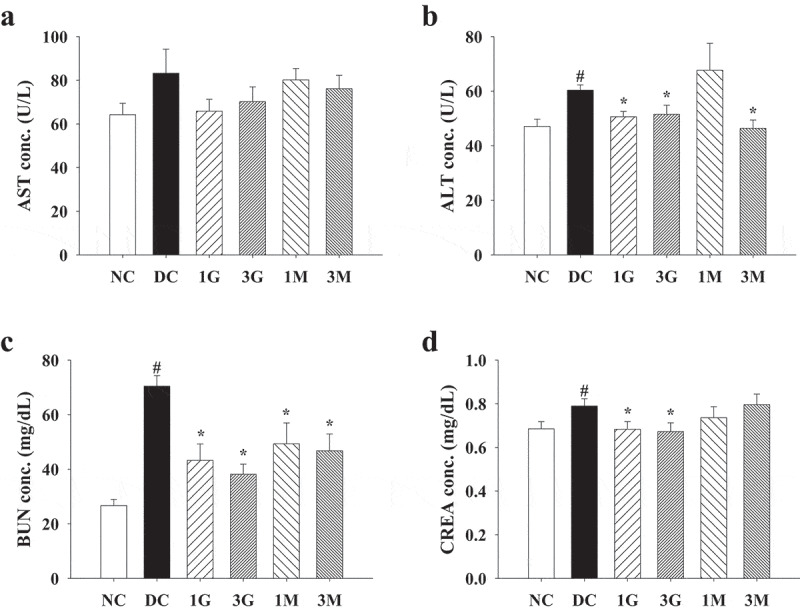
The concentrations of plasma (a) aspartate aminotransferase (AST), (b) alanine aminotransferase (ALT), (c) creatinine (CREA) and (d) blood urea nitrogen (BUN) in rats fed with *G. lucidum* powder supplement diets. The rats were injected with nicotinamide and STZ for the induction of diabetes and fed with *G. lucidum* powder supplement diets for 5 weeks. The blood samples were collected individually for the detection of plasma AST, ALT, CREA and BUN as described in Materials and methods. Results are expressed as mean ± SEM for each group of rats (n = 8). ^#^*p* < 0.05 compared with the NC group; *p < 0.05 compared with the DC group. NC: normal control; DC: diabetic control; 1 G: diabetic rats fed with 1% *G. lucidum* powders; 3 G: diabetic rats fed with 3% *G. lucidum* powders; 1 M: diabetic rats fed with 1% *G. lucidum* mycelium powders. 3 M: diabetic rats fed with 3% *G. lucidum* mycelium powders

**Figure 7.**
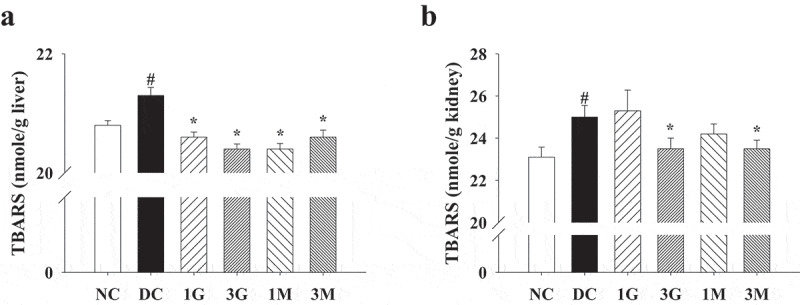
The concentrations of thiobarbituric acid reactive substances (TBARS) in the (a) liver and (b) kidney of rats fed with *G. lucidum* powder supplement diets. The rats were injected with nicotinamide and STZ for the induction of diabetes and fed with *G. lucidum* powder supplement diets for 5 weeks. The rats were then sacrificed to isolate the liver and kidney tissues. The concentration of TBARS in the liver and kidney was measured as described in **Materials and methods**. Results are expressed as mean ± SEM for each group of rats (n = 8). ^#^*p* < 0.05 compared with the NC group; *p < 0.05 compared with the DC group. NC: normal control; DC: diabetic control; 1 G: diabetic rats fed with 1% *G. lucidum* powders; 3 G: diabetic rats fed with 3% *G. lucidum* powders; 1 M: diabetic rats fed with 1% *G. lucidum* mycelium powders. 3 M: diabetic rats fed with 3% *G. lucidum* mycelium powders

As hyperglycaemia during diabetes is the pivotal mechanism leading to oxidative stress in tissues and organs, we finally investigated the antioxidant and protective effects of the *G. lucidum* supplement diets against diabetes-associated liver and kidney damage. Impaired liver and kidney functions were evidenced by increased plasma levels of ALT, CREA and BUN in diabetic rats compared with the levels in the NC group, although the level of AST was not significantly different between each group (Figure 6). The *G. lucidum* supplement diets significantly reduced ALT, CREA, and BUN levels (Figure 6). In addition, liver and kidney levels of TBARS, a marker for lipid peroxidation, were also reduced in rats fed with the *G. lucidum* supplement diets (Figure 7). These findings suggest the beneficial effects of the *G. lucidum* supplement diets on the improvement of impaired liver and kidney functions in diabetic rats, which was closely associated with its antioxidant activity.

## Discussion

Fungi and their metabolites are the main source of bioactive molecules that show biological activity (Girometta 2019; Ogbole et al. 2019). Among these sources, *G. lucidum* fruiting bodies have been reported to have antidiabetic activities. However, there is limited information on the hypoglycaemic and antioxidant effects of submerged *G. lucidum* cultures and the mechanism of action behind these effects. In the present study, we demonstrated the hypoglycaemic activity of whole submerged *G. lucidum* cultures and elucidated its potential mechanism in a rat model of type 2 diabetes. This notion has been substantiated by several lines of evidence. Firstly, dietary supplementation with 3 G culture for 5 weeks significantly reduced FPG and HOMA-IR levels in rats with type 2 diabetes. Secondly, OGTT showed a markedly reduced plasma glucose levels in diabetic rats fed with a 3 G supplement diet. Thirdly, an elevated liver glycogen level, enhanced hepatic hexokinase, G6PD, intestinal lactase and sucrase activity, and a lower G6Pase/hexokinase ratio were observed in rats fed with the *G. lucidum* supplement diet. Furthermore, diabetes-associated oxidative damage in the liver and kidney, as indicated by increased plasma levels of ALT, CREA, and BUN, was alleviated by *G. lucidum* supplementation. Our data clearly substantiate the hypoglycaemic and antioxidant activities of *G. lucidum* submerged cultures and provide critical insights into the mechanisms of action behind their effects.

*G. lucidum* fruiting bodies has been shown to exhibit hypoglycaemic activity. For example, consumption of the water extract of *G. lucidum* fruiting bodies (0.03 and 0.3 g/kg per day for 4 weeks) could decrease the plasma glucose levels through suppression of the hepatic PEPCK gene expression in db/db mice (Seto et al. 2009). The 30-day treatment with the hydroethanolic extract (containing beta-glucan, proteins, and phenols) of *G. lucidum* fruiting bodies exhibited hypoglycaemic effects in STZ-induced diabetic rats (Bach et al. 2018). In addition, many identified bioactive constituents in *G. lucidum* fruiting bodies, such as polysaccharides, proteoglycans, proteins, ganoderic acids, and triterpenoids, have been shown to exhibit hypoglycaemic activities (Ma et al. 2015). Polysaccharides inhibited hyperglycaemia by regulating the expression of several key enzymes in the glucose metabolism pathway, such as hepatic glucokinase, phosphofructokinase, G6PD, hepatic glycogen phosphorylase, fructose-1,6-bisphosphatase, phosphoenolpyruvate carboxykinase, and G6Pase (Xiao et al. 2012). *G. lucidum* proteoglycan could inhibit the activity of protein tyrosine phosphatase (PTP) 1B, a promising therapeutic target in diabetes, and ameliorated plasma glucose in db/db mice (Wang et al. 2012). In addition, decreased FPG was observed in rats with type 2 diabetes treated with the proteoglycan FYGL extracted from *G. lucidum* fruiting bodies. FYGL exerts its hypoglycaemic activity *via* the inhibition of PTP1B expression and activity (Teng et al. 2012). Moreover, treatment with submerged culture mycelium powder (1 g/kg/day) for 2 weeks reduced blood glucose and glycosylated haemoglobin concentrations in rats with type 1 diabetes (Vitak et al. 2015).

In the present study, a slightly lower plasma glucose level was observed in diabetic rats daily fed with a mycelia supplement diet compared with that in rats in the DC group. However, daily treatment with 3 G supplement diets significantly reduced plasma glucose levels, indicating that extracellular components may contain major compounds with hypoglycaemic activities and/or extracellular components exhibit a synergistic hypoglycaemic effect with mycelia. Polysaccharides are among the major extracellular components contained in whole submerged *G. lucidum* cultures, and polysaccharide intake was calculated as 36 and 107 mg/kg/day for rats in the 1 G and 3 G groups, respectively. Xiao et al. (2012) have reported that daily treatment with polysaccharides extracted from *G. lucidum* fruiting bodies (50–100 mg/kg) significantly decreases fasting serum glucose levels in rats with type 2 diabetes in a dose-dependent manner. Most importantly, the hypoglycaemic effect of polysaccharides has been associated with decreased expression of several key enzymes involved in gluconeogenesis and/or glycogenolysis (Xiao et al. 2012). Concordantly, our data also show enhanced hexokinase and G6PD activities and decreased G6Pase/hexokinase ratio in diabetic rats fed with a 3 G supplement diet. Accordingly, we suggest that extracellular polysaccharides harvested from submerged cultures and those extracted from fruiting bodies might exert their hypoglycaemic effects through the same mechanism of action.

Type 2 diabetes is an inflammatory metabolic disease that is commonly accompanied by increased oxidative stress. It has been reported that *G. lucidum* could reduce peroxide accumulation in the body by increasing antioxidant enzyme activity in the blood and liver (Jia et al. 2009). Both ethanol and water extracts of *G. lucidum* could reduce lipid peroxidation in the liver and kidney (Shieh et al. 2001). Supplementation with *G. lucidum* fruiting bodies polysaccharides decreased plasma CREA and BUN levels *via* the modulation of lipid peroxidation (He et al. 2006). We further demonstrated that supplementation with submerged *G. lucidum* cultures and their mycelia improved liver and kidney function potentially *via* the attenuation of oxidative stress. In the current study, the value of AST was comparable between each group. It is suggested that the oxidative stress elicited by the employed animal model was not violent enough to damage the mitochondria, since ALT and AST are critical enzymes prevalently located in the hepatocyte cytoplasm and mitochondria, respectively (Glinghammar et al. 2009; Jiang et al. 2015). Based on the results of the present study and previous studies, it could be speculated that polysaccharides are potential active components of *G. lucidum* with antioxidant activities and protective effects against liver and kidney damage.

## Conclusion

Our findings provide the first evidence of the dose-dependent hypoglycaemic activity of submerged *G. lucidum* cultures in type 2 diabetic rats, which has been closely associated with the modulation of carbohydrate digestion and metabolism. Antioxidant activities of submerged *G. lucidum* cultures potentially contribute to their protective effect against diabetes-associated liver and kidney damage. Because of the advantages of submerged cultures over basidiocarp cultivation and their promising hypoglycaemic and antioxidant activities, the products of submerged *G. lucidum* cultures could prospectively be developed as functional foods or additives for controlling type 2 diabetes.

## Data Availability

The data used to support the findings of this study are available from the corresponding author upon request.
